# Plasma Metabolic Signature of Atherosclerosis Progression and Colchicine Treatment in Rabbits

**DOI:** 10.1038/s41598-020-63306-y

**Published:** 2020-04-27

**Authors:** Mario Augusto Izidoro, Alberto Cecconi, María Isabel Panadero, Jesús Mateo, Joanna Godzien, Jean Paul Vilchez, Ángeles López-Gonzálvez, Jesús Ruiz-Cabello, Borja Ibañez, Coral Barbas, Francisco J. Rupérez

**Affiliations:** 1 Centro de Metabolómica y Bioanálisis (CEMBIO), Facultad de Farmacia, Universidad San Pablo-CEU, CEU Universities, Urbanización Montepríncipe, Boadilla del Monte, 28660 Madrid Spain; 2 Centro Nacional de Investigaciones Cardiovasculares (CNIC), Melchor Fernández Almagro, 3, 28029 Madrid, Spain; 3 IIS-Fundación Jiménez Díaz University Hospital, Av. de los Reyes Católicos, 2, 28040 Madrid, Spain; 4 CIBER de Enfermedades Cardiovasculares (CIBERCV), Instituto de Salud Carlos III, Monforte de Lemos 3-5, Pabellón 11, Planta 0, 28029 Madrid, Spain; 5 Center for Cooperative Research in Biomaterials (CIC biomaGUNE), Basque Research and Technology Alliance (BRTA), Paseo Miramon 182; 20014 Donostia/San Sebastián, Gipuzkoa, Spain; 6 IKERBASQUE, Basque Foundation for Science, Maria Diaz de Haro 3, 6 solairua, 48013 Bilbao/Bilbo, Bizkaia, Spain; 7 Universidad Complutense de Madrid, José Antonio Novais 12, Ciudad Universitaria, 28040 Madrid, Spain; 8 Associação Beneficente de Coleta de Sangue (Colsan), Av. Celso Garcia, 4815 - Parque São Jorge, São Paulo, SP 03063-000 Brazil; 9 Servicio de Cardiología, Hospital Universitario de La Princesa, Diego de León, 62, 28006 Madrid, Spain; 10 Clinical Research Centre, Medical University of Bialystok, ul.Jana Kilinskiego 1, 15-089 Białystok, Poland

**Keywords:** Metabolomics, Cardiovascular biology

## Abstract

Balloon catheter endothelial denudation in New Zealand white rabbits fed high cholesterol diet is a validated atherosclerosis model. Well-characterized in terms of atherosclerosis induction and progression, the metabolic changes associated with the atherosclerosis progression remain indeterminate. Non-targeted metabolomics permits to develop such elucidation and allows to evaluate the metabolic consequences of colchicine treatment, an anti-inflammatory drug that could revert these changes. 16 rabbits underwent 18 weeks of atherosclerosis induction by diet and aortic denudation. Thereafter animals were randomly assigned to colchicine treatment or placebo for 18 weeks while on diet. Plasma samples were obtained before randomization and at 36 weeks. Multiplatform (GC/MS, CE/MS, RP-HPLC/MS) metabolomics was applied. Plasma fingerprints were pre-processed, and the resulting matrixes analyzed to unveil differentially expressed features. Different chemical annotation strategies were accomplished for those significant features. We found metabolites associated with either atherosclerosis progression, or colchicine treatment, or both. Atherosclerosis was profoundly associated with an increase in circulating bile acids. Most of the changes associated with sterol metabolism could not be reverted by colchicine treatment. However, the variations in lysine, tryptophan and cysteine metabolism among others, have shown new potential mechanisms of action of the drug, also related to atherosclerosis progression, but not previously described.

## Introduction

Atherosclerosis is a chronic inflammatory disease that progressively leads to myocardial infarction and stroke^1^. Recruitment of monocytes within the vascular wall is an early phenomenon in the development of atherosclerotic plaques^2^. Its activation and transformation into macrophages release chemotactic molecules and proteolytic enzymes that develop and successively destabilize the plaques^3,4^.

Animal models of atherosclerosis have been laborious to ascertain since important hallmarks of human disease are not fully replicated in other species. Rabbits may provide valuable insights and allow the transfer of findings to understand human atherosclerosis and evaluate therapies for it because their lipid metabolism is rather like humans and unlike rodents.

Native New Zealand White rabbits (NZW) fed with a cholesterol-rich diet (1%) for at least 8 weeks represent the quickest way to establish arteriosclerosis^5^. It will mostly induce macrophage-rich fatty streaks, and the development of more complex atherosclerotic plaques (more similar to those of humans) might involve longer periods (from 6 months to several years) with lower proportion of cholesterol (0.2–0.75%)^6^ because the hypercholesterolemia (above 1 g/dL) achieved under so high cholesterol amount can lead to increased mortality due to hepatic toxicity^7^. The combination of high-fat/high cholesterol diet with angioplasty-induced aortic denudation of the aorta overcomes these drawbacks and provides a robust study model of human atherosclerosis and atherothrombosis^8^.

The characterization of the model is usually assessed through histological evaluation of the affected area, combined with the general determination of the lipid profile in the blood (cholesterol in lipoproteins)^9,10^.

Nevertheless, this model is far from being completely characterized, and the metabolic alterations associated with such a combination of diet and aortic endothelial denudation in rabbits have not been studied.

Diagnosis and prognosis of atherosclerosis and cardiovascular diseases is still a challenge, and Metabolomics (also known as metabolic phenotyping, profiling, or fingerprinting) offers a comprehensive, holistic possibility to study the changes in the metabolism that can be associated with the development of the atheromatous plaque in the context of a hypercholesterolemic diet, as well as to evaluate the metabolic impact of a new treatment^11^. Non-targeted (or untargeted) metabolomics workflow is aimed to unveil significant compounds that characterize one condition, as compared to control. Therefore, this methodological approach offers the possibility to discover which metabolic routes can be involved in the appearance or development of metabolic disturbances, which lead to cardiovascular complications. In addition, the compounds relevant in these routes can be proposed as diagnostic biomarkers of the condition under study^12^. Moreover, non-targeted metabolomics has been successfully applied to the study of human atherosclerotic plaques^13,14^, as well as to other animal models of atherosclerosis^15^.

Studies have demonstrated that platelet inhibitors or beta-blockers used in the treatment of ischemic cardiovascular disease, act primarily through inhibition of the atherosclerotic process at different pathophysiological stages^16,17^, but do not revert the process. Statins, also commonly used in both primary and secondary prevention of heart disease, show pleiotropic effects, which include stabilization of the plaque and reduction of inflammatory markers^18^.

Colchicine is an alkaloid extracted from *Colchicum autumnale* that acts as a ligand of tubulin, thus altering the process of polymerization of microtubules at the cellular level. In rheumatology, it is commonly used for the treatment of inflammatory diseases like gout, pseudogout, familial Mediterranean fever, and Behçet’s disease^19–21^. Interestingly, patients with gout or Mediterranean family fever treated with colchicine suffer from lower ischemic events than expected, together with lower mean platelet volume and beta-thromboglobulin, assuming that this could be a consequence of treatment with colchicine^22–24^.

Currently, colchicine is not indicated in the treatment of ischemic heart disease. However, advances in the knowledge of its pharmacodynamics^25–27^ and recent clinical trials^28,29^ indicate that it could be a precious ally in the prevention of cardiovascular complications.

The aim of this work was studying one animal model used in the study of drugs for ischemic heart disease treatment, and the mechanism of action of colchicine, through a multiplatform metabolomics approach to obtain broader metabolite coverage.

## Results & Discussion

**Figure 1:**
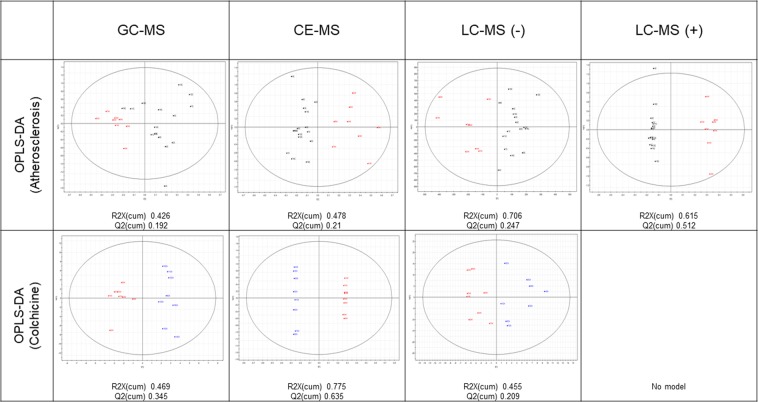
Scores plots from supervised MVA (OPLS-DA). Black squares: rabbits under hypercholesterolemic diet, before aorta denudation (18 weeks); Blue circles: at the end of the study (36 weeks) without colchicine; Red diamonds: at the end of the study (36 weeks), receiving colchicine.

Each analytical platform (GC-MS, CE-MS, LC-MS(+) and LC-MS(−)) gave one matrix of compounds and samples, including QCs. PCAs for each platform are shown in Supplementary Figure 1. The clustering of the QC samples permits us to consider the analytical conditions as reproducible. Therefore, the differences between samples can be associated with the sample class. Nevertheless, PCAs show only small differences between samples/groups, and the supervised MVAs (OPLS-Das, Fig. 1) were also tested. The OPLS-DA models were not strong and satisfactory enough to highlight the compounds significant for the classification, which lead to the conclusion that the observed differences between groups are too slight to be captured through MVA.

To find significantly different metabolites, two-way repeated-measures ANOVA was applied. The factors tested were “atherosclerosis progression”, as associated with time, and “colchicine treatment”, together with the interactions between both factors. 54 metabolites were found statistically significant (Table 1) and annotated.

**Table 1:** Multiplatform metabolomics metabolite coverage. Annotated significant (p < 0.05) compounds per platform, factor, and biochemical class, as found after repeated measurements two-way ANOVA.

Platform	Total	Atherosclerosis progression	Colchicine treatment	Interaction	Biochemical class
GC-MS	7	5	3	4	Glucose derivatives (1), short chain organic acids (4), amino acids and derivatives (1), sterols (1)
CE-MS	9	8	2	1	Amino acids and derivatives (9)
LC-MS(−)	18	17	5	1	Bile acids (11), sterols (4), other lipids (3)
LC-MS(+)	20	18	6	1	Amino acids and derivatives (1), bile acids (9), sterols (5), other lipids (4)
Sum	54	48	16	7	

Our MS-multiplatform metabolomics approach has permitted us to show up variations in metabolites from several biochemical classes: glucose derivatives, central carbon metabolism, amino acids and their derivatives, sterol metabolism (bile acids and other sterols), and lysophospholipids. Most of the significant compounds (38 out of the 54) were found with LC-MS (both positive and negative), and most of the significant variations were associated with the time-course evolution of atherosclerosis (48 out of the 54). It is noteworthy that, in the context of advanced atherosclerosis for all animals, we have been able to find 16 metabolites that were significantly different due to the treatment of colchicine. Moreover, we were able to find 7 compounds that showed an interaction between both factors, *i.e*., the influence of each factor on the abundance of the metabolite is not independent of that of the other factor, and the result is different from the mere addition or subtraction due to each factor.

**Figure 2:**
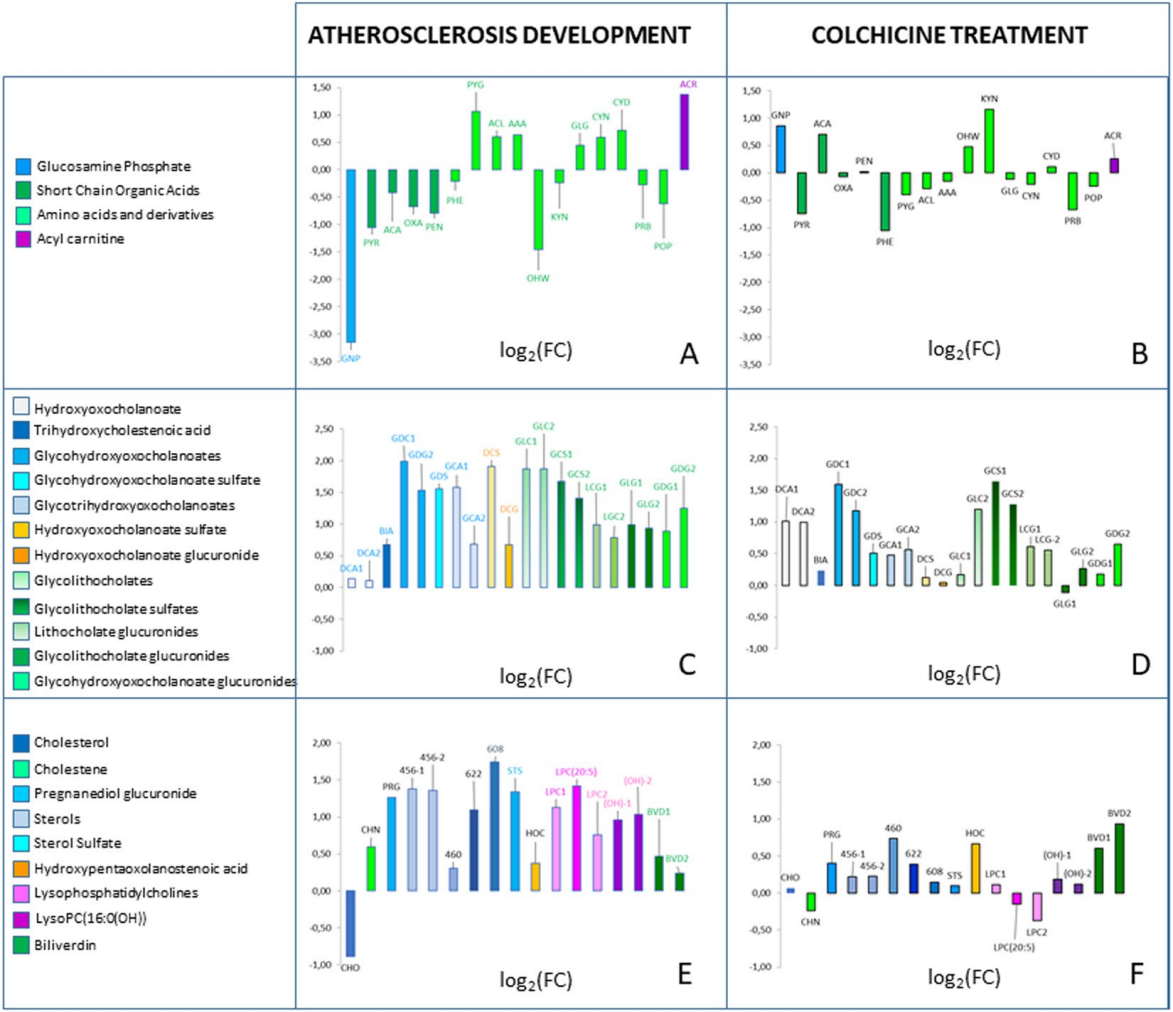
Graphical summary of the variations. All metabolites that were significantly changed due to the factor Atherosclerosis, or the factor Colchicine, or both are gathered. Ordinates represent log_2_(FC) between the compared groups: Pannels (A,C,E), changes 36 weeks vs. 18 weeks, without colchicine (atherosclerosis progression); Pannels (B,D,F), changes 36 weeks with colchicine vs. 36 weeks without colchicine. Codes for metabolites as in Table 2.

Detailed results for each metabolite are shown in Table 2, where they are sorted according to their biochemical class. The logarithm to base 2 of the fold change – log_2_(FC) – associated with each factor (atherosclerosis progression or colchicine treatment), are graphically presented also in Fig. 2.

**Table 2:** Summary of the quantitative variation of all annotated metabolites that were found significant in any of the comparisons, grouped by the family of compounds. 36W-ATH: 36 weeks atherosclerosis without colchicine treatment; 18 W: 18 weeks atherosclerosis; 36W-CCH, 36 weeks with colchicine treatment.

Biochemical class	Compound names	CODE	Technique	ID level^50^	36W-ATH *vs*. 18 W log_2_FC	36W-CCH *vs*. 36W-ATH log_2_FC	FACTOR: ATHEROSCLEROSIS p-value	FACTOR: COLCHICINE p-value	FACTORS INTERACTION p-value
Glucose derivative	Glucosamine 1-phosphate	GNP	GC/MS	2	−3.15	0.86	3.3·10^−02^	NS	1.1·10^−02^
Short-Chain Organic Acids	Pyruvic acid	PYR	GC/MS	2	−1.06	−0.74	2.2·10^−03^	9.6·10^-03^	NS
	Acetoacetate	ACA	GC/MS	2	−0.42	0.71	NS	NS	3.1·10^−02^
	Oxalic acid	OXA	GC/MS	2	−0.67	−0.07	2.2·10^−03^	3.5·10^−02^	NS
	Pentanoic acid	PEN	GC/MS	2	−0.79	0.02	3.4·10^−02^	1.3·10^−02^	2.9·10^−02^
Amino acids and amino acids derivatives	Phenylalanine	PHE	LC/MS(+)	2	−0.21	−1.05	4.4·10^−02^	8.7·10^−03^	NS
	Pyroglutamine	PYG	CE/MS	3	1.07	−0.40	3.0·10^−03^	NS	NS
	Acetyl-Lysine	ACL	CE/MS	2	0.60	−0.29	4.0·10^−04^	NS	NS
	2-Aminoadipic acid	AAA	CE/MS	2	0.64	−0.15	4.7·10^−03^	NS	NS
	Glycyl-Glutamate	GLG	CE/MS	2	0.44	−0.12	8.9·10^−03^	NS	NS
	5-hydroxy-tryptophan	OHW	GC/MS	2	−1.46	0.48	NS	NS	2.0·10^−02^
	Kynurenine	KYN	CE/MS	2	−0.23	1.16	9.0·10^−04^	4.9·10^-04^	2.1·10^−04^
	Cystathionine	CYN	CE/MS	2	0.59	−0.21	2.4·10^−03^	NS	NS
	Cystine	CYD	CE/MS	2	0.72	0.11	2.5·10^−03^	NS	NS
	Proline betaine	PRB	CE/MS	3	−0.28	−0.67	NS	1.7·10^−02^	NS
	Prolylhydroxyproline	POP	CE/MS	3	−0.62	−0.25	1.6·10^−02^	NS	NS
	Acyl Carnitine	ACR	LC/MS(+)	3	1.37	0.26	9.2·10^−05^	NS	NS
Bile acids	Glycohydroxyoxocholanoate [glycol deoxy/chenodeoxy/ursodeoxy cholate]	GDC-1	LC/MS(−)	2	1.99	1.59	4.3·10^−02^	4.2·10^−02^	NS
	Glycohydroxyoxocholanoate [glycol deoxy/chenodeoxy/ursodeoxy cholate]	GDC-2	LC/MS(+)	2	1.53	1.18	9.7·10^−03^	2.6·10^−02^	NS
	Glycohydroxyoxocholanoate glucuronide [glyco deoxy/chenodeoxy/ursodeoxy cholate glucuronide]	GDG-1	LC/MS(+)	3	0.88	0.18	7.8·10^−03^	NS	NS
	Glycohydroxyoxocholanoate glucuronide [glyco deoxy/chenodeoxy/ursodeoxy cholate glucurornide]	GDG-2	LC/MS(−)	2	1.25	0.65	1.3·10^−05^	NS	NS
	Glycohydroxyoxocholanoate sulfate [glyco deoxy/chenodeoxy/ursodeoxy cholate sulfate]	GDS	LC/MS(−)	3	1.56	0.51	1.4·10^−04^	NS	NS
	Glycolithocholate	GLC-1	LC/MS(−)	3	1.87	0.17	4.4·10^−03^	NS	NS
	Glycolithocholate	GLC-2	LC/MS(+)	3	1.87	1.20	1.8·10^−02^	NS	NS
	Glycolithocholate glucuronide	GLG-1	LC/MS(−)	3	0.99	−0.11	2.3·10^−03^	NS	NS
	Glycolithocholate glucuronide	GLG-2	LC/MS(−)	2	0.94	0.27	6.1·10^−04^	NS	NS
	Glycolithocholate sulfate	GCS-1	LC/MS(−)	3	1.67	1.64	3.8·10^−02^	4.7·10^-02^	NS
	Glycolithocholate sulfate	GCS-2	LC/MS(+)	2	1.41	1.28	4.6·10^−02^	3.6·10^−02^	NS
	Glycotrihydroxyoxocholanoate [glycocholate]	GCA-1	LC/MS(+)	2	1.58	0.47	4.2·10^−05^	NS	NS
	Glycotrihydroxyoxocholanoate [glycocholate]	GCA-2	LC/MS(+)	2	0.68	0.56	8.0·10^−06^	NS	NS
	Hydroxyoxocholanoate [deoxy/chenodeoxy/ursodeoxy cholate]	DCA-2	LC/MS(+)	3	0.11	0.99	3.9·10^−02^	3.5·10^−03^	NS
	Hydroxyoxocholanoate [deoxy/chenodeoxy/ursodeoxy cholate]	DCA-1	LC/MS(−)	3	0.14	1.01	NS	4.9·10^−02^	NS
	Hydroxyoxocholanoate glucuronide [deoxy/chenodeoxy/ursodeoxy cholate glucuronide]	DCG	LC/MS(+)	3	0.68	0.04	1.5·10^−03^	NS	NS
	Hydroxyoxocholanoate sulfate [deoxy/chenodeoxy/ursodeoxy cholate sulfate]	DCS	LC/MS(−)	3	1.91	0.12	6.9·10^−03^	NS	NS
	Lithocholate glucuronide	LCG-1	LC/MS(−)	2	0.99	0.61	4.6·10^−05^	4.9·10^−02^	NS
	Lithocholate glucuronide	LCG-2	LC/MS(−)	3	0.78	0.56	1.4·10^−03^	NS	NS
	Trihydroxycholestenoic acid/Dihydroxyoxocholestanoic acid [cholate]	BIA	LC/MS(+)	3	0.67	0.23	1.5·10^−04^	NS	NS
Sterols (non bile acids)	Cholestene	CHN	GC/MS	2	0.60	−0.24	3.9·10^−05^	NS	NS
	Cholesterol	CHO	LC/MS(+)	3	−0.89	0.07	NS	1.6·10^−02^	NS
	Hydroxypentaoxolanostenoic acid	HOC	LC/MS(−)	2	0.38	0.67	1.0·10^−02^	4.2·10^−02^	NS
	Pregnanediol glucuronide	PRG	LC/MS(−)	3	1.26	0.40	6.6·10^−05^	NS	NS
	Sterol Sulfate	STS	LC/MS(+)	3	1.34	0.10	4.7·10^−04^	NS	NS
	Sterol-456	STE-456-1	LC/MS(−)	3	1.38	0.22	2.1·10^−04^	NS	NS
	Sterol-456	STE-456-2	LC/MS(+)	3	1.36	0.22	3.0·10^−04^	NS	NS
	Sterol-460	STE-460	LC/MS(+)	3	0.30	0.74	NS	4.8·10^−02^	NS
	Sterol-608	STE-608	LC/MS(−)	3	1.74	0.15	2.1·10^−04^	NS	NS
	Sterol-622	STE-622	LC/MS(+)	4	1.09	0.39	5.3·10^−03^	NS	NS
Other lipids	LysoPC(16:0(OH))	LPC(OH)−1	LC/MS(+)	3	0.96	0.19	7.5·10^−03^	NS	NS
	LysoPC(16:0(OH))	LPC(OH)−2	LC/MS(+)	3	1.03	0.12	7.5·10^−03^	NS	NS
	LysoPC(20:5)	LPC(20:5)	LC/MS(−)	3	1.42	−0.15	4.1·10^−04^	NS	NS
	Lysophosphatidylcholine	LPC-1	LC/MS(−)	2	1.13	0.11	1.9·10^−04^	NS	NS
	Lysophosphatidylcholine	LPC-2	LC/MS(−)	3	0.76	−0.38	3.1·10^−02^	NS	2.8·10^−03^
	Biliverdin	BVD-1	LC/MS(+)	3	0.47	0.61	3.9·10^−03^	NS	NS
	Biliverdin	BVD-2	LC/MS(+)	3	0.24	0.93	3.0·10^−03^	NS	1.9·10^−02^

### Central carbon and amino acids metabolism

In Fig. 2A,B metabolites found significant, mainly from GC-MS and CE-MS, have been grouped. They correspond to metabolites related to the central carbon metabolism (short-chain organic acids) amino acid metabolism, and one compound from the hexosamine pathway (glucosamine phosphate).

The increase in glucosamine phosphate (GNP) is related to the atherosclerotic lesion. In endothelial cells, alterations in the metabolism of GNP have been associated with atherosclerosis and other pathological manifestations^30^. In Table 2, it can be seen that the signal associated with this metabolite in rabbits that did not receive colchicine was lower at 36 wk. On the contrary, those rabbits that received the colchicine showed at 36 wk higher levels of GNP than those not treated. Although the colchicine factor was not significant, the *p*-value associated with the interaction was lower than 0.05. In this experimental model where there is atheroma plaque but not hyperglycemia, the treatment could be enhancing the activity of glutamine:fructose-6-phosphate amidotransferase (GFAT) in endothelial cells^31^, therefore modulating the angiogenesis and the vulnerability of the plaque^32^.

Regarding amino acid metabolism, we found differences associated with atherosclerosis but not to the colchicine in the glutamine and lysine pathways. Pyroglutamine was higher in our model of atherosclerosis at the end of the study, although not significant alteration was detected in the levels of glutamine. The role of this metabolite is not clear, but its relationship with atherosclerosis and Incident Heart Failure risk has been shown up in the Atherosclerosis Risk in Communities (ARIC) Study^33,34^. Acetyl-lysine and aminoadipic acid levels in plasma are related to the extent of post-translational modifications (PTMs) of proteins, and both were increased due to the combination of diet and arterial denudation. Lysine acetylation/deacetylation is a process known to occur only in proteins, one of the most important PTMs of histones and other proteins^35^. Therefore, its variations in plasma could be useful to infer the rate of such processes. In particular, histone deacetylation has been attributed to a role in the occurrence of atherosclerosis through several processes^36^. Also, 2-aminoadipic acid was higher at the end of the study, which could be related to the higher activity of myeloperoxidase in the atherosclerotic lesion^37^.

Phenylalanine and two metabolites related to tryptophan metabolism (5-OH-tryptophan and kynurenine) showed a significant response to the colchicine treatment. In the case of phenylalanine, the treatment enhanced the decrease in circulating phenylalanine, whereas colchicine counteracted the decrease of both tryptophan metabolites. Although phenylalanine variations are known to be associated to atherosclerosis, this association is not clearly elucidated: concentration of phenylalanine was decreased in the urine of atherosclerotic rats^38^, whereas in plaques from patients undergoing carotid endarterectomy it was found associated to the age of the patient and the levels of low-density lipoproteins (LDL)^39^.

5-hydroxytryptophan is an intermediate in the serotonin pathway, an alternative to the main tryptophan degradation route, the kynurenine pathway. The ratio Kynurenine/Tryptophan has been correlated to the activity of IDO, indoleamine 2,3 dioxygenase, which is increased in atherosclerosis-related complications^40^. Our model only partially fits with this alteration, because it was not possible to find significant differences associated to the progression of atherosclerosis neither in kynurenine nor in tryptophan, but colchicine induced higher levels of both metabolites, and therefore the interaction between both factors became significant.

The Gly-Glu dipeptide was found increased and associated with atherosclerosis progression. Although not previously related to the formation of the plaque, it has been proven that it is a neurotrophic factor for acetylcholinesterase^41^. Inhibition of such activity attenuates atherogenesis in another animal model of atherosclerosis, the ApoE knockout mice^42^.

### Sterols metabolism

The most prominent variation that could be associated with the progression of atherosclerosis was the marked increase in bile acids and sterol metabolism-related compounds (Table 2, Fig. 2C,D). Cholesterol levels were found decreased at the end of the study, as compared to the intermediate point (18 weeks of hypercholesterolemic diet) because bile acids inhibit 7α-hydroxylase, the key enzyme in the synthesis of bile acids. Primary and secondary bile acids were higher, which was partially expected because cholesterol-fed rabbits accumulate bile acids in plasma to a higher extent than other mammals^43^, and the atherogenic effect is related to the capacity of the animal to eliminate cholesterol as bile acids. Moreover, although bile acid sequestration could be a therapeutic strategy, as demonstrated in mice, colchicine has not proven any positive effect in this metabolic pathway, and variations of bile acids associated with colchicine (most of them non-significant) were enhancing the increase due to atherosclerosis progression.

## Conclusions

Animal models of disease are still essential to improve our understanding of complex alterations of the healthy state, and to study the mechanisms of action of new treatments. Besides the alterations in the plaque, our results show that atherosclerosis progression in rabbits under high cholesterol diet and after balloon catheter endothelial denudation, was clearly characterized by dramatic changes in the profile of sterol-related compounds.

Overall, we have applied for the first time one multiplatform metabolomics approach to evaluate the biochemical changes associated to the development of the atheroma plaque in the rabbit model of atherosclerosis.

With this methodology, the biochemical impact of one pharmacological treatment of potential interest can be evaluated. We tested colchicine, an anti-inflammatory with potential interest in the treatment of cardiovascular complications. Although colchicine was not able to revert the changes associated to atherosclerosis progression, we could find changes, not previously described, in other metabolic routes. This permits to expand the knowledge about the processes associated with such treatment, in the context of atherosclerosis progression.

## Materials and Methods

### Animal treatment

Experimental atherosclerosis was induced in male NZW rabbits bred by a combination of a high cholesterol diet (0.2%) and balloon endothelial denudation of the abdominal aorta^8^: A Fogarty catheter is inserted through femoral access and progresses to the middle third of the descending thoracic aorta. The balloon is inflated with angiographic contrast to document the position of the balloon by fluoroscopy. Careful removal is performed to denudate the intima of the aorta without causing further damage. It is denuded from the descending thoracic aorta to near the abdominal aorta bifurcation. The process is repeated 3 times. The Fogarty catheter is then removed, and the femoral artery is completely ligated. This complete ligation does not cause major ischemia in the lower limb since rabbits have a highly developed collateral circulation system.

The animals were of similar age (3 months) and weight (3.1 ± 0.3 kg) at the beginning of the study. They began with a standardized diet for rabbits on arrival at the National Center for Cardiovascular Research (CNIC) for 2 weeks to adapt to the new environment, then the hypercholesterolemic diet was initiated and maintained for the 36 weeks of the study. The abdominal aortic denudation procedure was performed two weeks after starting the high cholesterol diet. More severe atheroma lesions are achieved in the abdominal aorta portion. After induction of the atherosclerotic plaques (at 18 weeks of study), blood samples were taken. The animals were then randomly distributed in 2 groups with equal plaque load: Group I (n = 10) received a daily injection s.c. of 1 ml saline whereas Group II (n = 10) received a daily injection of 0.2 mg/kg colchicine s.c. for 18 weeks. At week 36 a second blood sample was taken; then the animals were sacrificed. Two rabbits died during the pre-randomization phase secondary to vascular complications of arterial access for the denudation procedure, while two rabbits died between weeks 18 and 36. Therefore, 16 rabbits (8 per group) completed the whole experiment. The CNIC Ethics Committee for Animal Experimentation (ECAE) approved all procedures (registration number PROEX 164/15), according to the Spanish laws (Law 32/2007 of 7 November; Royal Decree 53/2013 of 1 February) and European directive 2010/63/EU.

Plasma samples collected from the rabbits were stored, aliquoted and prepared for the analysis in three different chromatographic platforms. Before analysis, Quality Controls (QCs) were prepared for each analysis platform by pooling 50 µL of each sample and subjecting them to the same metabolite extraction and derivatization procedures as the other samples of the sequence.

#### Instrumental analysis

Sample treatment and analysis for all techniques were performed as described elsewhere^44–47^. All procedures and conditions are fully described in the Supplementary material.

#### Data treatment

##### LC-MS and CE-MS signal processing

The resulting data obtained from the LC-MS and CE-MS analysis were cleaned of background noise and unrelated ions by the Molecular Feature Extraction (MFE) tool provided by MassHunter Profinder software as described elsewhere^47^. Briefly, a list of possible components that represents the full TOF mass spectral data feature is generated. This list is a collection of co-eluting ions, related by charge-state, isotopic distribution and/or different adducts, neutral losses and dimmers present. For the data extraction, several parameters were set to the applied algorithm, making 250 counts as the limit for the background noise. Then all features were aligned across all the samples files using mass and RT to build a unique spectrum for each compound group and enabling the re-extraction of the batch files, the Recursive Feature Extraction. This recursive analysis uses the list of ions generated by the MFE algorithm to perform target data mining with Find by Ion (FbI) algorithm to minimize the number of missing values and therefore improve the quality of the data.

### Compound identification from LC-MS and CE-MS

The statistically significant compounds, selected as described below, were putatively annotated by searching their accurate mass against public databases i.e., METLIN (http://www.metlin.scripps.edu), KEGG (http://www.genome.jp/kegg), LIPIDMAPS (http://www.lipidmaps.org) and HMDB (http://www.hmdb.ca), all simultaneously accessed through an in-house developed search engine, CEU Mass Mediator (http://ceumass.eps.uspceu.es/mediator)^48,49^. This annotation corresponds to level 3 of metabolite identification according to Schrimpe-Rutledge *et al*.^50^.

For compounds included in our in-house libraries, retention/migration times were also compared, and this adds more confidence to the annotation, as to be assigned level 2 of metabolite identification. Besides, for deeper annotation of some statistically significant features from LC-MS analysis, an MS/MS experiment was set up, and the fragmentation pattern was analyzed. In some cases, this permitted to increase the Level of identification from level 3 to 2, whereas in others, only some characteristic fragments were found, and the feature was assigned to a biochemical class, but full elucidation was not possible.

### Signal processing and compound identification for GC-MS

The data obtained by the GC-MS analysis was processed and treated with several Agilent Technologies tools as described previously^47^. Deconvolution and metabolite identification were performed by the MassHunter Unknown Analysis Tool 7.0. This software searched against two libraries (Fiehn library v2008 and the CEMBIO in-house spectral library for plasma) to assigned chemical identities to the compounds by comparing both RT and extracted spectra during the deconvolution against each compound included in the library. The commercial library NIST (National Institute of Standards and Technology) library 2 v2014 was used to annotate non-identified compounds: compounds with spectrum similarity score >80% and concordant Retention Index (*n*-alkene scale) were putatively identified. The matrix obtained was then aligned with the MassHunter Profiler Professional (MPP) B.12.1. Signal integration and assignment of target ions were performed by MassHunter Quantitative Analysis B.06.00. (Agilent Technologies). To minimize analytical drift normalization to the IS signal was performed.

### Data quality assurance

Data quality was assured by using the QC samples as reported before^51,52^. Briefly, raw data from all samples (subject and QCs samples) were processed as described above for each analytical technique. Then, data were filtered by using the QC group as reference: features present in 100% of QC samples with a coefficient of variation below 30% were retained. The final data matrix underwent PCA in SIMCA P + 12.0.1 software to test the analytical reproducibility of the data^53^.

### Statistical analysis

Univariate statistical analysis (UVA) was carried out with an in-house developed script for MATLAB R2015 (Mathworks, Inc., Natick, MA, USA) whereas SIMCA P + 12.0.1 was used for multivariate analyses (MVA). For MVA, log-transformation and Unit Variance (UV)-scaling were used to build the respective matrices per technique. Unsupervised (Principal Components Analysis, PCA) and supervised (orthogonal partial least squares discriminant analysis, OPLS-DA) analyses were applied to check trends, outliers, and to select discriminating variables correspondingly. For UVA, Repeated Measures ANOVA was applied. The factors were the development of atherosclerosis (“within factor”, associated with time) and the treatment with colchicine (“between factor”, associated with the group). Bonferroni-corrected *p*-values for the significance of each factor, as well as for the interaction, were computed for all metabolites. Fold change (FC) was calculated as (average value in the case group/average value in the reference group). For those metabolites that showed *p*-value < 0.05 for any factor or interaction, two log_2_(FC) were calculated: of the comparison of [rabbits after 36 weeks *vs*. rabbits after 18 weeks, both without colchicine] (*i.e*., atherosclerosis progression) and [rabbits after 36 weeks with colchicine *vs*. rabbits after 36 weeks without colchicine] (*i.e*., colchicine treatment).

## Supplementary information

Supplementary information.

Supplementary information2.

## Data Availability

The datasets generated during and/or analysed during the current study are available from the corresponding author on reasonable request.
