# Supplementary figures and images for: Plasma Metabolic Signature of Atherosclerosis Progression and Colchicine Treatment in Rabbits
Source: Sci Rep. 2020 Apr 27;10:7072. doi: 10.1038/s41598-020-63306-y (PMC7184732; doi:10.1038/s41598-020-63306-y)

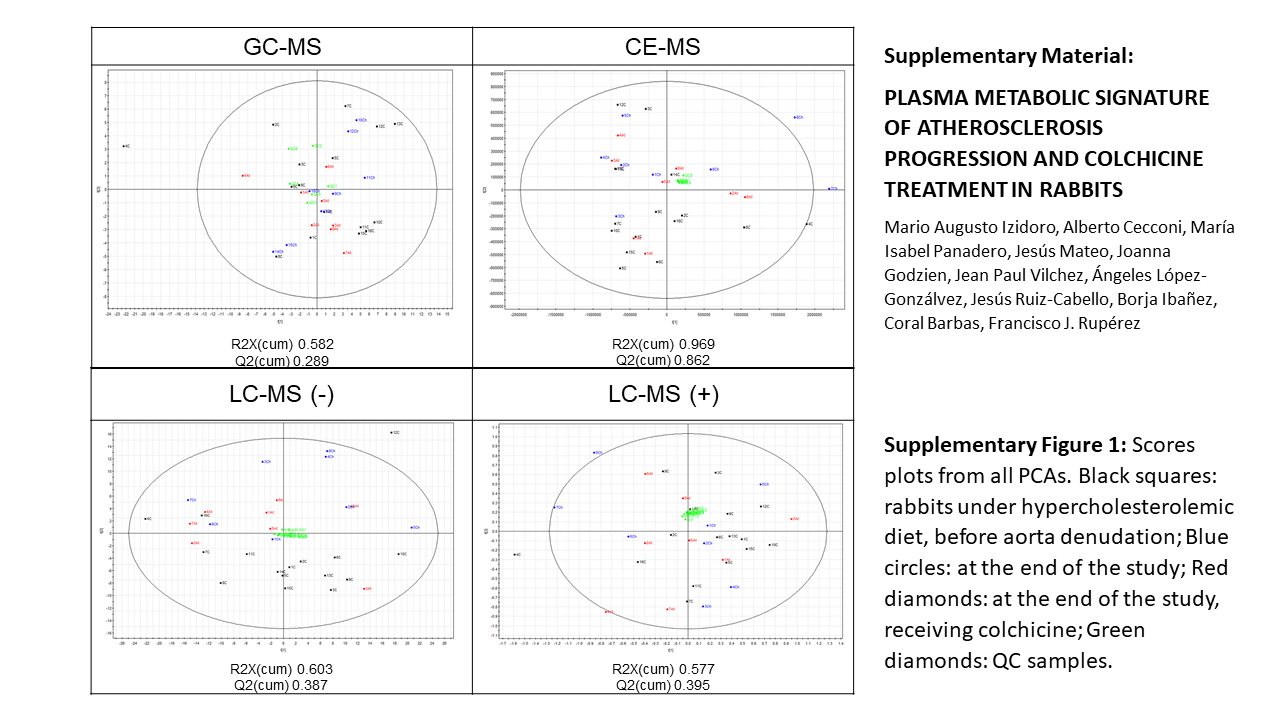

Supplement: Supplementary file 1 — Supplementary information. [file 41598_2020_63306_MOESM1_ESM.tif]
