# Supplementary material for: Plasma Metabolic Signature of Atherosclerosis Progression and Colchicine Treatment in Rabbits
Source: Sci Rep. 2020 Apr 27;10:7072. doi: 10.1038/s41598-020-63306-y (PMC7184732; doi:10.1038/s41598-020-63306-y)
Supplement: Supplementary file 2 — Supplementary information2. [file 41598_2020_63306_MOESM2_ESM.docx]

Supplementary Material:

PLASMA METABOLIC SIGNATURE OF ATHEROSCLEROSIS PROGRESSION AND COLCHICINE TREATMENT IN RABBITS

Mario Augusto Izidoro, Alberto Cecconi, María Isabel Panadero, Jesús Mateo, Joanna Godzien, Jean Paul Vilchez, Ángeles López-Gonzálvez, Jesús Ruiz-Cabello, Borja Ibañez, Coral Barbas, Francisco J. Rupérez

## MATERIALS & METHODS

### Instrumental analysis

#### Chemicals and reagents

All the aqueous solutions and the LC-MS mobile phase were prepared using ultrapure water obtained “in-house” from a Milli-Qplus 185 system (Millipore, Billerica, MA, USA). LC-MS grade acetonitrile and analytical grade formic acid were purchased from Fluka Analytical (Merck-Sigma-Aldrich Chemie, Stenheim, Germany). All derivatization grade chemicals used in the GC-MS analysis (O-methoxyamine hydrochloride and N,O-bis(trimethylsilyl)trifluoroacetamide BSTFA) respectively, were obtained from Merck - Sigma-Aldrich Chemie. The internal standard (L-methionine sulfone) used in the CE-MS analysis was also purchased from Merck-Sigma-Aldrich Chemie.

#### LC-MS analysis

Plasma samples preparation and analysis was performed as previously described[1]. Briefly, for deproteinization 100 µL plasma was vortex-mixed with 300 µL of a chilled mixture of methanol/ethanol (1:1), followed by the centrifugation at 4 °C and 15000 *g* for 20 minutes for the pellet formation. The supernatant was collected carefully and transferred to the analysis LC-MS vials.

The experiments were performed in a LC-QTOF-MS with a liquid chromatography (LC) system (1200 series, Agilent Technologies, Waldbronn, Germany) consisting of a degasser, two binary pumps, thermostated autosampler (maintained at 4 °C) and a column oven (at 60 °C), coupled to an ESI QTOF (6520 Agilent Technologies) with a scan rate of 1.02 scans/s operated in full scan mode from 50-1000 *m/z*. Capillary voltage was 3500 V for positive ion mode and -4500 V for negative ion mode, nebulizer gas flow rate was 10 L/min, source temperature at 350 °C and pressure of 40 psi. Two reference masses (*m/z* 121.0509 (C_5_H_4_N_4_) and *m/z* 922.0098 (C_18_H_18_O_6_N_3_P_3_F_24_)) were continuously injected into the system for positive ion mode (ESI +) and two (*m/z* 112.9856 (C_2_O_2_F_3_) and *m/z* 966.0007 (C_18_H_18_O_6_N_3_P_3_F_24_·HCOOH)) for negative ion mode (ESI -) to guarantee constant mass correction. Separation was achieved by using a Discovery HS C18 column (150 x 2.1 mm, 3.5 µm) with a Discovery HS C18 (20 x 2.1 mm, 3 µm) guard column, both from Supelco (Bellefonte, MA USA). 10 µL from each sample were injected onto the column with a flow rate of the mobile phase (A: water with 0.1% formic acid and B: acetonitrile with 0.1% formic acid) of 0.4 mL/min running in a gradient from 5 to 100% B.

#### CE-MS analysis

For the CE-MS analysis, each plasma sample (100 µL) was mixed with 100 µL of formic acid (0.2 M) and 5% of acetonitrile and methionine sulfone (0.4 mM) as internal standard. Samples were vortex-mixed for 1 minute and then transferred to a Centrifree Millipore (30 kDa) filter and centrifuge at 2000 *g*, 70 minutes at 4 °C. The supernatant was directly transferred to the analysis vials[2].

The analysis was carried out optimizing a previously reported method[3] with a 7100 CE system coupled through ESI source to a 6224 TOF-MS system. A 1200 series ISO Pump was used to supply the sheath liquid. Every piece of equipment was from Agilent Technologies. For separation, a fused-silica capillary (125 cm x 50 μm, Agilent Technologies) was employed. A new capillary was conditioned with NaOH (0.1 M) for 30 minutes, and 30 minutes of ultrapure water. Before each analysis, the capillary was flushed for five minutes (950 mbar) with background electrolyte solution (BGE, 0.8 M formic acid in 10% methanol). the sheath liquid was infused along with the samples at flow rate of 0.6 mL/min and with 1/100 split. The sheath liquid composition was methanol/water (1/1, v/v), containing 1 mmol/L formic acid with two reference masses (*m/z* 121.0509 and 922.0098). Samples were injected for 35 seconds with 50 mbar; then, to improve repeatability, BGE was co-injected along with the samples for 20 seconds at 100 mbar. The separation conditions included: 25 mbar of pressure, 30 kV of voltage with a resultant 20 μA current, for a total separation time of 35 min. Before each injection the instrument automatically replaced the BGE. Data were collected in ESI positive ion mode on a system operated in full scan mode (*m/z* 85-1000) with a scan rate of 1 scan/s. The drying gas flow rate was 10 L/min, nebulizer 10 psi, voltage 3500 V, gas temperature 200 °C, fragmentor 100 V, and skimmer 65 V. The total separation time was divided into two segments: the first minute without applying nebulization and the remaining minutes with nebulization. BGE and sheath liquid were freshly prepared and degassed by sonication for 5 min prior to use in order to ensure proper and reproducible ionization.

#### GC-MS analysis

For GC-MS analysis, 100 µL of plasma was vortex-mixed with 300 µL of acetonitrile to guarantee deproteinization, and then centrifuged for 10 minutes at 15400 *g* at 4 °C. 100 µL of the supernatant was transferred to the GC-MS analysis vials containing glass inserts, then the solvent was evaporated with a Savant™ SPD131DDA SpeedVac™ (ThermoFisher Scientific, Waltham, MA USA) at 30 °C. For methoximation, 10 µL of O-methoxyamine hydrochloride (15 mg/mL) in piridine was added to each analysis vial and vortexed. Then the vials were covered and incubated in the darkness at room temperature for 16 hours. Then 10 µL of BSTFA with 1% TMCS (v/v) were added, the samples were vortexed and incubated at 70 °C for 1h for silylation reaction. At the end of the procedure, 100 µL of Internal Standard C18:0 methyl esther (10 mg/mL in heptane) was added to each GC vial. Three blank samples were prepared by following the same procedure for extraction and derivatization. These blanks were analyzed at the beginning, in the middle and at the end of the sequence[4].

Samples were analyzed as reported[5, 6], with a GC system (7890B) coupled with a Q-TOF-MS (7250) from Agilent Technologies. For the separation, an aliquot of 2 µL was loaded onto a J&W guard column (10 m x 0.25 mm, 0.25 μm, Agilent Technologies) integrated with a DB5-MS column (30 m x 0.25 mm, 0.25 μm film, Agilent Technologies). The sample was injected in split mode with a split ratio of 1/10 with 3−10 mL/min helium flow. Helium carrier gas was employed at constant flow rate of 1 mL/min. The column temperature was initially maintained at 60 °C for 1 min and then increased at the rate of 10 °C/min to reach a final temperature of 325 °C, and before cool-down held for 10 min. Temperatures of injector, transfer line, filament source and the quadruple were maintained at 250, 290, 230 and 150 °C, respectively. The system operated in full scan mode (*m/z* 50-600) at a rate of 2 spectra/s, and with the EI set at -70eV. Then, a retention time locked (RTL) method was applied to reduce the retention time (RT) variation of the whole analysis. One n-FAMEs solution was run for retention index determination.

References

[1] M. Ciborowski, F. Javier Ruperez, M. Paz Martinez-Alcazar, S. Angulo, P. Radziwon, R. Olszanski, J. Kloczko, C. Barbas, Metabolomic Approach with LC-MS Reveals Significant Effect of Pressure on Diver's Plasma, Journal of Proteome Research. 9 (2010) 4131-4137, doi: 10.1021/pr100331j.

[2] S. Naz, A. Algaba Calderon, A. Garcia, J. Gallafrio, R. Teijeiro Mestre, E. Gonzalez Gonzalez, d.C. Munoz, M.C. Martin Delgado, J.A. Lorente Balanza, A.V. Colnaghi Simionato, N. Nin Vaeza, C. Barbas, F.J. Ruperez, Unveiling differences between patients with acute coronary syndrome with and without ST elevation through fingerprinting with CE-MS and HILIC-MS targeted analysis, Electrophoresis. 36 (2015) 2303-2313, doi: 10.1002/elps.201500169.

[3] G.A. Canuto, E. Castilho-Martins, M.F. Tavares, L. Rivas, C. Barbas, Á López-Gonzálvez, Multi-analytical platform metabolomic approach to study miltefosine mechanism of action and resistance in Leishmania, Anal Bioanal Chem. 406 (2014) 3459-3476, doi: 10.1007/s00216-014-7772-1.

[4] M. Vallejo, A. Garcia, J. Tunon, D. Garcia-Martinez, S. Angulo, J. Martin-Ventura, L. Blanco-Colio, P. Almeida, J. Egido, C. Barbas, Plasma fingerprinting with GC-MS in acute coronary syndrome, Analytical and Bioanalytical Chemistry. 394 (2009) 1517-1524, doi: 10.1007/s00216-009-2610-6.

[5] T. Kind, G. Wohlgemuth, D.Y. Lee, Y. Lu, M. Palazoglu, S. Shahbaz, O. Fiehn, FiehnLib: Mass Spectral and Retention Index Libraries for Metabolomics Based on Quadrupole and Time-of-Flight Gas Chromatography/Mass Spectrometry, Anal. Chem. 81 (2009) 10038-10048, doi: 10.1021/ac9019522.

[6] A. Mastrangelo, A. Ferrarini, F. Rey-Stolle, A. Garcia, C. Barbas, From sample treatment to biomarker discovery: A tutorial for untargeted metabolomics based on GC-(EI)-Q-MS, Anal. Chim. Acta. 900 (2015) 21-35, doi: 10.1016/j.aca.2015.10.001.
